# Rating of Perceived Exertion as a Method to Determine Training Loads in Strength Training in Elderly Women: A Randomized Controlled Study

**DOI:** 10.3390/ijerph18157892

**Published:** 2021-07-26

**Authors:** Carlos Leandro Tiggemann, Caroline Pietta-Dias, Maira Cristina Wolf Schoenell, Matias Noll, Cristine Lima Alberton, Ronei Silveira Pinto, Luiz Fernando Martins Kruel

**Affiliations:** 1 School of Physical Education, Centro Universitário da Serra Gaúcha, Caxias do Sul 95.020-472, Brazil; 2 Universidade do Vale do Taquari, Lajeado 95.914-014, Brazil; 3 Exercise Research Laboratory, School of Physical Education, Physiotherapy and Dance, Universidade Federal do Rio Grande do Sul, Porto Alegre 90.690-200, Brazil; carolpieta@yahoo.com.br (C.P.-D.); mairacws@yahoo.com.br (M.C.W.S.); ronei.pinto@ufrgs.br (R.S.P.); kruel@esef.ufrgs.br (L.F.M.K.); 4 Goiano Federal Institute of Education, Science and Technology, Ceres 76.300-000, Brazil; matiasnoll@yahoo.com.br; 5 Department of Sports Science and Clinical Biomechanics, University of Southern Denmark, 5230 Odense, Denmark; 6 Department of Sports, School of Physical Education, Federal University of Pelotas, Pelotas 96.055-630, Brazil; cristine.alberton@ufpel.edu.br

**Keywords:** RPE, self-selected load, perception of effort, muscle strength, resistance training, resistance exercise, intensity

## Abstract

**Objective**: The aim of this study was to compare the effects of training using loads from a repetition maximum value (%1RM) and rating of perceived exertion (RPE) in elderly women. **Methods**: Twenty-five elderly women (60–75 years old) were randomly assigned to a group that trained using loads determined by 1RM test (G%; *n* = 12) or to a group that trained using loads determined by RPE (GPE; *n* = 13). Elderly women trained for 12 weeks using five exercises performed with 2–3 sets of 8–15 repetitions. Loads progressed from 45% to 75% of 1RM (G%) and from 13 to 18 from Rating Perceived Exertion of Borg Scale (GPE). The outcome measures, 1RM and maximum repetitions (RMs with 70% 1RM), were assessed before, between and after training programs. **Results**: Increased 1RM value and RMs were observed in both groups (20–42%, *p* < 0.001 and 56–76%, *p* < 0.001, respectively, for %G; and 17–56%, *p* < 0.001 and 47–106%, *p* < 0.001, respectively, for GPE), without differences between them. **Conclusions**: Prescribing loads using the RPE and 1RM might be similarly effective for training elderly women in order to promote strength gains. As a practical application, RPE could be an additional method to determine training loads. In spite of the promising results of the present study, it is not possible to state that the use of RPE is effective in monitoring loads during sub maximal strength training in elderly and more research must be carried out to confirm it.

## 1. Introduction

Strength training has been shown to be effective in improving health in elderly population, potentially via increases in maximum strength, muscle power and muscular endurance [1]. Furthermore, strength training can promote increases in muscle mass, bone mineral density, and joint mobility [2]. These benefits may lead to a reduced risk of falls and improvements in functional activities of daily life, significantly contributing to improving quality of life in this population [2,3].

In order to achieve optimal results via strength training, it is critical to properly select the loads to be used in training, given loads are one of the key aspects for a proper training prescription [4]. The methods of prescribing loads are based on a percentage of one repetition maximum (%1RM) or repetitions maximum ranges (RMs) [5]. An alternative method for monitoring the load during strength training is the use rating of perceived exertion (RPE), which can be defined as the subjective intensity of effort, strain, discomfort, and/or fatigue that is experienced during aerobic and strength exercises [6]. It has been shown that similar loads can be achieved using RPE compared to traditional methods [7] with recommendation for use with healthy adults [8], elderly and heart disease patients [9]. However, the effectiveness of using RPE in training load monitoring has not been determined by experimental studies.

For elderly population, few studies using RPE in strength training were found. Tomporowski et al. [10] used RPE to assess training intensity after sets, but not to prescribe training loads. Vincent et al. [11] used RPE as a supporting measures to increment training load in sets of different exercises. Barret and Smerdely [12] used RPE between 11 and 13 (light and somewhat hard in Borg’s scale) in the initial stage of the training (two initial sessions), and evolved to RPE between 15 and 17 in latter 18 sessions (hard/heavy and very hard). Bean et al. [13] set training intensity with RPE between 11 and 16, whereas De Vreede et al. [14] opted for the Borg 10 points Scale and controlled effort between 7 and 8 (very strong). Likewise, Nelson et al. [15] used only RPE during trunk flexions and extensions (RPE 16), and Tiggemann et al. [1] used RPE in the power training prescription for elderly women, with intensities ranging from 13 to 18 in the Borg RPE scale.

However, it is important to highlight that none of the previous studies aimed at comparing different approaches for monitoring training intensity (% of 1RM and RPE, for example). Although it has been found strong correlations between the RPE and the amount of load used in a strength training [7], these data are based on cross-sectional study, without information on how the load progression would behave when that is modulated by RPE. Two important aspects should be highlighted: (1) the suggestions presented in the literature, for example in the recommendations suggested by the ASCM in its official position [16], for the choice of subjective load in strength training is not based on experimental studies, but on a theoretical construct, and also, (2) the second aspect is the fact that the effective use in RPE in chronic studies with strength training are scarce, and what actually occurs is the use of acute responses predicting a possible chronic response [16].

To address these gaps, the aim of this study was to compare the effects of strength training using loads prescribed by 1RM or RPE in elderly women. The hypothesis of this study was that similar gains should be observed from elderly women exercising using either 1RM or RPE. We chose to evaluate elderly people in this study due to the concern related to the increase of elderly people in our country and in the world, and it is essential to find effective training strategies for this audience. Moreover, we have selected specifically women population to participate in our study due to greater accessibility to this audience in the city where the study was conducted, as well as studies by our research group have noticed a greater adherence to training in this profile of subjects. In addition, different studies have found a similar behavior of perceived exertion in strength exercises between men and women [17,18].

## 2. Methods

### 2.1. Experimental Approach to the Problem

Twelve weeks of strength training were conducted with elderly women randomly allocated to two groups. They were trained using five exercises performed with 2–3 sets of 8–15 repetitions. Loads progressed from 45% to 75% of 1RM (G%) and from 13 to 18 RPE (GPE). Identical training volumes and intensities were used for both groups. Our study follows the CONSORT guidelines (Consolidated Standards Of Reporting Trials) [19].

### 2.2. Subjects

**Figure 1:**
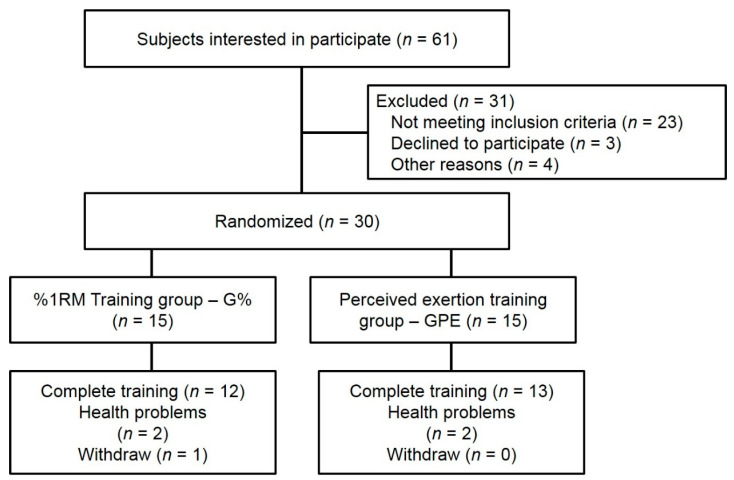
Flowchart with description of subject recruitment and assignment to training groups.

Sample size was estimated using the GPower v 3.1 program opting by a α level of 5%, power of 90% and effect sizes from previous studies [20], leading to a minimum of twelve subjects per group. Recruitment for this study was held in the city of Caxias do Sul (Brazil) through advertisements in social networks, local newspapers and radio stations, and the website of the Institution where the study was conducted (Faculty of Serra Gaúcha–FSG). From advertisement, 61 women made contact with 31 being excluded (4 were absent in the initial meeting for clarification, 3 due to incompatibility in schedules, and 24 did not meet the inclusion criteria (see Figure 1)). We selected just women to participate in our study due to greater accessibility to this audience in the city where the study was conducted, and studies by our research group have noticed a greater adherence to training in this profile of subjects.

Ten subjects were not included due to chronic diseases (heart disease, arthritis, diabetes, fibromyalgia) and six due to performing physical exercises twice of three times per week. Three subjects were not included because they did not have medical agreement for taking part of the exercise program; three performed strength training in the last six months, and two were out of the age range for the study (60–75 years). By drawing numbers, subjects were either allocated to the group that used loads determined by 1RM value (G%) or to the group that trained with loads determined by RPE (GPE).

From subjects’ demographics, there were no significant differences between groups for body mass, height, body mass index (BMI) and age at baseline (week 0; Table 1). Adherence to training sessions was above 98% (range 91–100%), the level of physical activity of the subjects was rated as moderate or high [21]. Self-assessment of health (using 0–10 scale) indicated good health status (Table 1). The most often chronic diseases from participants at baseline were hypertension, dyslipidemia, and diseases associated with thyroid and osteoarthritis.

### 2.3. Procedures

This study was defined as a randomized clinical trial with longitudinal assessment and non-probabilistic sampling. Ethics approval was granted from the Local Ethics Committee (registration number 22108) in accordance with the Declaration of Helsinki, and registration was granted at the Brazilian Registry of Clinical Trials under code RBR-8qkx34 (http://www.ensaiosclinicos.gov.br/rg/RBR-8qkx34/) (accessed on 20 December 2020). All subjects were carefully informed of the design of the study and gave their written consent to participate. Subjects of the study and researchers who took independent measurements were blinded from subject allocation to both groups during training, while the technicians who supervised training sessions inevitably had group knowledge. Same equipment and tools, locations and hours of collection have been reproduced in different evaluations.

**Figure 2:**
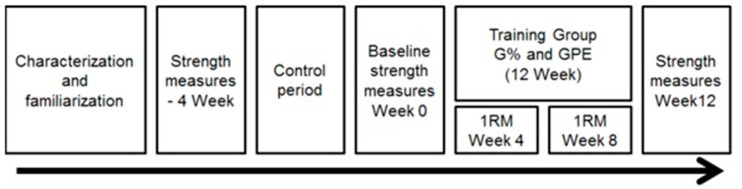
Timeline of the experimental procedures. Note: G%—group that trained using loads determined by 1RM; GPE—group that trained using loads determined by perceived exertion (RPE).

This study started at a stage characterization and familiarization followed by stages of strength assessment, a control period, and finally a training period followed by assessments of strength (see Figure 2).

### 2.4. Characterization and Familiarization Stages

Prior to the assessments of strength preceding the control period, all subjects underwent an interview involving medical history, completion of the International Physical Activity Questionnaire validated in Brazil [21], assessment of body mass (using a measuring scale, Filizola, São Paulo, Brazil), height (using a stadiometer, WCS, Curitiba, Brazil), and determination of BMI (body mass/heigth^2^).

Two familiarization sessions consisting of 2 sets of 15 repetitions for each exercise commonly used in strength training (horizontal leg press, bench press, leg curl, seated row, and leg extension, Ajustfitness, Caxias do Sul, Brazil) were performed. A controlled pace of 2 s for each phase was used (concentric and eccentric; using a digital metronome, Qwik Time, Beijing, China). The minimum load for each exercise was used in the first set, following adjustments using RPE between 13 and 15, which leads to ~50% of 1RM in sedentary adult subjects [7]. Changes in load of 5–20% were performed during each set in order to achieve the desired RPE.

Familiarization with RPE scale followed [7]: (1) a presentation of the perceived exertion scale (Borg RPE 15 points, 6–20) [22] reading guidelines for using Borg RPE scale suggested by Gearhart et al. [23], adapted for this study; (2) definition of muscle localized RPE, which according to Lagally et al. [24] is specific to the muscle groups and joints involved in a given exercise; (3) definition of reference values for minimum and maximum RPE using the method of memory or recall suggested by Robertson and Noble [6] and Gearhart et al. [23]. The minimum effort should elicit rating 6 (absence of any effort) while RPE rating 19 should elicit maximum effort required to perform maximum repetitions (extremely hard). RPE rating 20 (maximum exertion) should be felt only in hypothetical cases, not being used as a reference [22]. The same criteria were used at various times and recalled during training sessions. The choice of RPE scale was made because this scale show good correlations between ratings and loads in strength training [7], and good relation with other traditional scales with ten ratings [25].

### 2.5. Maximum Dynamic Strength

For the assessment of the maximum dynamic strength, the test of 1RM was performed for all exercises used in training program. The test involved completing a single repetition of range of motion with maximum load following recommendations from Brown and Weir [26] and protocols used previously [7]. 1RM evaluations were applied at weeks −4, 0, 4, 8, and 12.

### 2.6. Muscular Endurance

Muscular endurance assessment was conducted using 70% load values of 1RM bench press and knee extension in order to measure the maximum number of repetitions performed during exercise. Local muscle endurance was operationally defined as the ability to resist muscle fatigue when using submaximal resistance [27], that is, a load lower than maximum strength (1RM). In this way, muscular endurance can be expressed either on an absolute or relative basis. Absolute muscular endurance involves performing a set with as many repetitions as possible at a fixed load [28] and in this case, 70%1RM.

A series of repetitions was performed, until failure, using 70% of 1RM of the respective exercise, with the same load being used in the pre- and post-training phases. Exercise pace was controlled in order to cover each phase (concentric and eccentric) in 2 s using feedback from a digital metronome. The same test was applied at 0 and 12 weeks using the load corresponding to the pre training session (absolute load).

### 2.7. Strength Training Programs

Training programs were conducted for 12 weeks, two times per week. The initial portion of each session consisted of a brief aerobic warm-up in a treadmill (5 min walk, 3–4 km h^−1^) and unloaded joint motions (with warm-up purposes). The main part involved the performance of five exercises (horizontal leg press, bench press, leg curl, seated row and knee extension), following trunk flexions. Training volume and intensity evolved using linear periodization. Volume was controlled by changing the number of sets and repetitions in each exercise and was similar between training groups (Table 2). Each micro cycle consisted of 2 weeks, in which each pair of microcycles involved a mesocycle. Resting intervals between sets and exercises was approximately 2 min, and exercise pace was controlled in order to cover each phase (concentric and eccentric) in 2 s.

Training load for G% group was determined from the 1RM value (from 45 to 70%), whist training load for GPE was determined by RPE (from 13 to 18), with load adjustments in each micro cycle (Table 2). The initial GPE loads were defined based on the familiarization sessions prior to the training period. Linear periodization was a methodological choice by the authors, aiming to start with lighter loads, considering a sedentary sample, and evolving to a more robust load over the weeks. The selected intensities were based on the ACSM recommendations [16] for beginners and elderly people (40–50% 1RM), and based on these percentages, corresponding RPE values were adopted, according to previous cross-sectional studies) [1,7].

The RPE was evaluated at the end of the last set for each exercise. Whenever RPE was within the intended bandwidth (intended ± 1), load was not changed. If RPE was not achieved, 2.5–5% changes in load were conducted by the professionals in the following training session (5% for each rating perceived exertion outside the goal, with 0.25 kg of resolution). As an example, if intended RPE from meso cycle 2 was 15 ± 1, and RPE was 13, a 10% increase in load was conducted in the following training session. The option for these training intensities for the GPE group was based on previous relationship between RPE and %1RM tests in a previous cross-sectional study [29]. Whenever participants were unable to complete exercise series, RPE 19 was recorded, and the load was reduced by 10% (apart from the last series).

All participants in both groups performed the same testing procedures, and RPE was recorded after each exercise for both groups in order to mask assignment for subjects and technicians involved in training monitoring. Training load from each meso cycle was latter converted into percentages from the 1RM tests conducted at weeks 0, 4 and 8.

### 2.8. Statistical Analysis

Data were assessed using descriptive statistics (mean ± standard deviation) after analysis of normality in distribution and homogeneity tested by Shapiro–Wilk and Levene tests, respectively. Paired samples t tests were used to compare 1RM load results at the control period (weeks −4 and 0) within each group. For between groups comparison at baseline (week 0), independent samples t test was employed. In order to compare load from 1RM and RMs tests during the study time (weeks 0, 4, 8 and 12), a repeated measures ANOVA with group factor was employed (2 groups × 4 testing sessions) with post hoc correction of Bonferroni whenever main effects or interactions where significant. Significance differences were assumed when *p* ≤ 0.05, and all tests were conducted in a statistical package (SPSS 26.0, Inc., Chicago, IL, USA).

## 3. Results

Twenty-five subjects completed the study (G% = 12 and GPE = 13), and among the dropouts, one occurred due to incompatibility of time (changing jobs), four due to health issues (one fall, one varices, one pneumonia, and one pain in the neck), without any association with training.

During the control period (weeks −4 to 0), small changes in maximum strength were found for the leg press exercise in G% (from 62.2 ± 12.1 to 64.6 ± 12.0 kg, *p* = 0.03), and knee flexion exercise (from 13.4 ± 3.2 to 14.3 ± 3.1 kg, *p* = 0.03) and seated row (from 42.8 ± 6.1 to 41.1 ± 5.7 kg; *p* < 0.01) for the GPE. No differences were observed for all other exercises.

Table 3 shows group results for 1RM and RPE of all exercise sessions and each micro cycle. The values of both groups ranged between 45% and 73% in the 1RM, while RPE varied between 13 (somewhat hard) and 17 (very hard). The intention of this study was that groups perform training under maximum effort, yet some sets occurred to the concentric failure (RMs). Therefore, assessing the amount of exercise sets in which maximum repetitions were observed (RMs), subjects from G% performed only 37 (1.21%) of total 3060 sets, while in the GPE subjects performed 140 (4.22%) of the total 3315 sets.

**Figure 3:**
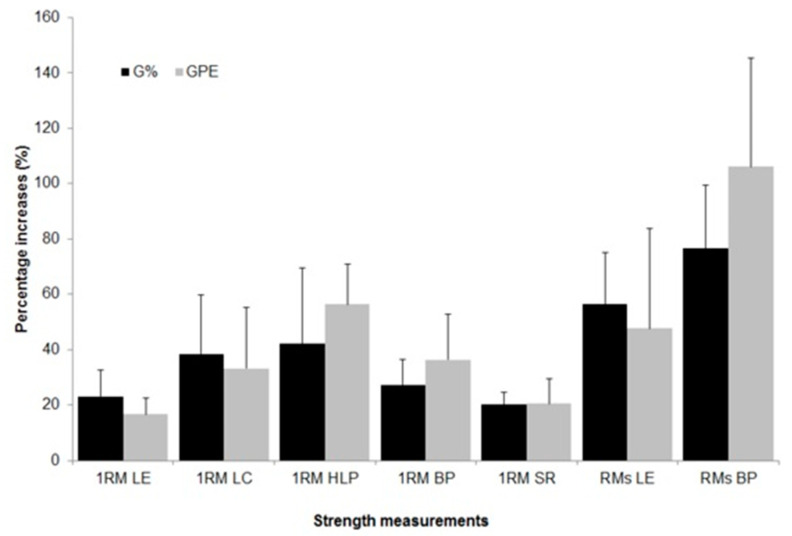
Percentage changes in maximum dynamic strength tests (1RM) and muscular endurance tests (RMs) for leg extension (LE), leg curl (LC), horizontal leg press (HLP), bench press (BP), and seated row (SR), in both training groups (G% and GPE).

Table 4 shows the results of tests of the 1RM for five exercises with significant increases between each evaluation session (*p* < 0.004), with similar responses between groups throughout training (*p* > 0.05). For RMs tests, there were significant increases between weeks 0 and 12 (*p* < 0.001), with no significant differences between groups (Table 4). For results from RMs tests in bench press, significant interaction was found between groups and training sessions, which was rejected when assessing main effects in separate (*p* > 0.05). The average percentage increases in 1RM tests and RMs are shown in Figure 3.

## 4. Discussion

The main results of this study demonstrated that strength training loads prescribed using RPE in elderly women lead to increases of 1RM (17–56%) and muscular endurance (47–106%). These gains in strength were similar to those observed in the training group using loads prescribed by the 1RM test, 20–42% in maximal strength and 56–76% in muscular endurance, similar to the magnitude of responses found in other studies [5,30].

For 1RM gains, Bottaro et al. [5] found increases of ~26% in the 1RM bench press and leg press for elderly men who underwent strength training for 10 weeks with loads of 40–60% 1RM. For muscular endurance, De Vos et al. [30] found improvements of 70% in bench press and 99% in knee extension exercise for a group that underwent training with 50% 1RM; however, the load used for assessment was 90% of 1RM. The increments in force observed in this short period of time can be justified by the large potential for adaptation in the elderly [31].

In our study, loads ranging between ~45 and 73% of 1RM were used, which may be considered low to moderate [32]. Recent meta-analysis showed that the use of low loads (<50% 1RM) produces similar gains in strength and hypertrophy in untrained subjects [33]. In contrast, others meta-analyses suggested that high intensity training (loads ~80% 1RM) results in larger gains in maximum strength [32,34]. Although this evidence indicates consistent results, it should be noted that, maximum strength gains are largely associated with greater loads. However, lower volumes (weekly frequency and number of sets) [35] and lower intensities (percentage of one repetition maximum; % 1RM) also provided consistent gains in strength, and in some cases, of the same magnitude [36]. Similarly, Hunter and Treuth [36] found negative significant correlations between increases in strength and the loads used, i.e., for smaller loads (50–60% 1RM) greater strength gains were found after 16 weeks of strength training.

Studies with longer interventions could possibly present similar results. Taaffe et al. [37] submitted two groups of elderly women to strength training with different loads (40 vs. 80% 1RM) for 52 weeks. For knee extensors, increases in strength were only observed for the group who trained with larger loads in the three initial months, without differences between groups after that. For other exercises (leg press and knee flexor), increments in strength were similar during all training programs. Even though higher intensities are more effective, lower intensities may be sufficient to improve maximum strength, especially when elderly have some limitation in sustaining high intensities [38]. Another important aspect to be considered in our study is that the majority of sets performed by subjects (>95% of the sets) did not follow a maximum repetition profile. Studies indicate that the number of repetitions to be performed should elicit concentric contraction failure at the last repetition [39]. For strength increases, motor units should be progressively fatigued, to the point that new motor units need to be recruited, predominantly type II which are prone to greater strength gains hypertrophy [40].

The use of maximum repetitions is questioned by some researchers [41,42,43]. In this issue, although not applied to elderly subjects, several studies have been conducted to compare the responses in training using maximum repetitions (concentric failure) to training with similar loads without maximum effort [44,45]. In the study from Folland et al. [45], both groups (failure and not failure) performed strength training for 9 weeks for knee extensors involving, for the maximum repetition group, 4 sets of 10 reps (~75% of 1RM), with 30 s of resting intervals. The other group (not failure) performed 40 sets of 1 rep (75% 1RM) with similar resting intervals. There were similar gains in maximal dynamic strength for both groups (1RM, 34% and 40% for the maximum and non-maximum repetition groups, respectively) and maximal isometric strength (18% and 15% for the maximum and non-maximum repetition groups, respectively). In the present study, the amount of maximum efforts (RMs) for both groups was small (less than 5% of sets) and yet significant increases in maximal strength were found after 12 weeks of training. Possibly individual responses can help to explain these results, i.e., elderly women with reduced initial strength in our study.

Other factors could be added to refrain from using maximum repetitions in strength training, like the preference of the subject by practicing exercise at lower intensities. Glass and Stanton [46] found that young subjects tend to opt for moderate load (~56%1RM) and reduced number of repetitions (~9) during strength training group. Along with that, using high training loads could induce to larger dropouts in physical exercise programs [47]. In line with that, the use of maximum effort for prolonged periods of training could possibly be associated with a high risk of overtraining and overuse injury in subjects with high physical fitness syndrome [42].

In this issue, RPE may be an alternative approach for monitoring strength training intensity, because it shows strong correlation to training loads [7,48], and it is a method of easy implementation and reduced time expenditure for application, refraining from maximum efforts. Although the use of RPE has been suggested as a tool for training monitoring different populations [8,9,49,50], it is important to state that these recommendations were not based on experimental studies that tested the effectiveness of RPE. Although the promising results of the present study, more clinical trials must be performed to confirm if it is possible to state that the use of RPE is effective in monitoring loads during sub maximal strength training in the elderly.

Few studies using RPE during strength training were found for elderly [1,10,13]. All studies found significant gains in strength; however, none of them aimed at comparing the responses of RPE with the traditional training method [12,13]. In the present study, the GPE had their training loads determined only by RPE, which were similar to the group that trained with loads determined by 1RM (Table 3). For RPE, similar results were found among training groups where RPE 13 (somewhat hard) represented an approximate load of 50% of 1RM, RPE 15 (hard) was approximately 65% of 1RM, and RPE 17 (very hard) was approximately 70% of 1RM. These load approximations have led to similar training gains without need for performing maximum efforts. Tiggemann et al. [1] performed a power training and a traditional training (moderate speeds) in elderly women with loads varying between 13 and 18 of the Borg Scale (load ranging from 45.1 to 75.5% of 1RM). The results indicated similar and significant increases (*p* < 0.05) in maximal strength (≈58% in leg press exercise, ≈19% in knee extensor exercise), power (≈30% in squat jump, ≈25% in counter movement jump) and functional capacity (range of 6.8 and 20% at performed tests).

It is important to note that the results of this study are limited to elderly subjects and women population, and although they were physically active, they often have decreased levels of muscle strength compared with young population. Sub-maximal exercise intensity in this population may not represent similar efforts limiting potential improvements in stronger subjects. The same issue may apply to longer training programs or other goals, such as maximum muscle hypertrophy. Moreover, as limitation, training was of short duration, with the highest and recommended loads being used only halfway through the end of the study. We highlight those future studies should investigate also more in-depth men populations.

Low-intensity initial loads have been indicated in the strength training, especially for beginners and elderly subjects, including, it appears, that important gains in muscle strength can be acquired, even when using loads of lower intensities (50% 1RM). In addition, a wide discussion has been held about the effective need to perform maximum efforts (understand the use of maximum repetitions–up to concentric failure) for the benefits related to maximum strength and hypertrophy [28,43]. The official position of the American College Sports Medicine [16] clearly suggests the use of perceived exertion in TF in the elderly: “Older individuals initiating a resistance training regimen, may begin with lower resistance, perhaps 40–50% of 1RM (i.e., very light to light intensity)”. However, its indications do not come from original studies that have effectively tested the effectiveness of the use of RPE in this modality and specific audience. Thus, seeking appropriate strategies for this audience is essential, and in this sense, the perception of effort must be tested and can be with a method of monitoring intensity, in which, through estimates, it is possible to avoid the performance of maximum efforts (repetitions with maximum loads) or the use of maximum loads (1RM test). Even though future studies are needed, the present work made a relevant contribution to the literature.

## 5. Conclusions

We conclude that the use of RPE might be effective in monitoring loads during sub maximal strength training in elderly women, promoting significant gains in muscle strength with similar benefit from training using loads prescribed by 1RM value, confirming our hypothesis.

Using the Borg RPE scale (15 points, 6–20) at the end of each strength exercise set represents an effective way of monitoring and adjusting loads during strength training programs. Progressive increases in loads can be sufficient to secure significant increases in muscle strength. The use of RPE can be an alternative to 1RM tests and maximum repetitions in subjects with low levels of muscular strength, thus optimizing the time spent during training prescription and avoiding strenuous efforts in the early stages. In spite of the promising results of the present study, it is not possible to state that the use of RPE is effective in monitoring loads during sub maximal strength training in elderly women at the moment. More research must be carried out to confirm it.

## Figures and Tables

**Table 1:** Subjects’ demographics (mean ± standard deviation—SD).

	G% (*n* = 12) Mean ± SD	GPE (*n* = 13) Mean ± SD	*p* Value
Body mass (kg)	70.2 ± 8.5	65.2 ± 8.9	0.167
Height (cm)	157.6 ± 4.9	155.8 ± 5.0	0.351
BMI (kg.m^−2^)	28.3 ± 3.7	26.8 ± 3.1	0.291
Age (years)	66.4 ± 4.5	65.6 ± 5.4	0.691
Training frequency (%)	98.1 ± 2.3	99.0 ± 2.0	0.340
Self-assement health (0 a 10)	8.3 ± 1.1	8.3 ± 1.0	0.965
IPAQ–rating (%)			
Sedentary	16.7	23.1	
Moderately active	75.0	53.8	
Very active	8.3	23.1	
Participation in support groups (%)	58.3	69.2	

Note: G%—group that trained using loads determined by 1RM; GPE—group that trained using loads determined by perceived exertion. No significance differences were observed (*p* > 0.05).

**Table 2:** Training volumes and intensities for G% (% 1RM) and GPE (Rating Perceived Exertion).

Meso	Micro	Week	SET × REP	%1RM for G%	RPE for GPE
1	1	1–2	2 × 15	45	13 ± 1
	2	3–4	2 × 15	50	14 ± 1
2	3	5–6	2 × 12	55	15 ± 1
	4	7–8	2 × 12	60	16 ± 1
3	5	9–10	3 × 8	65	17 ± 1
	6	11–12	3 × 8	70	18 ± 1

Note: Meso = meso cycle; Micro = micro cycle; SET x REP = sets and repetitions; %1RM = one repetition maximum percentage; RPE = perceived exertion.

**Table 3:** Mean ± standard deviation of the load as percentage of a single repetition maximum (%1RM) and as Perceived Exertion (RPE) for all exercises and sessions of each micro cycle.

Micro Cycle	G%		GPE	
	%1RM	RPE	%1RM	RPE
1	45.1 ± 0.3	12.5 ± 1.1	45.7 ± 3.7	12.5 ± 1.0
2	50.1 ± 0.2	13.0 ± 1.1	51.6 ± 9.2	13.3 ± 1.0
3	55.1 ± 0.3	13.5 ± 0.9	60.4 ± 4.6	13.9 ± 0.8
4	60.0 ± 0.3	14.1 ± 1.0	68.8 ± 9.9	16.3 ± 0.7
5	65.1 ± 0.3	14.6 ± 1.1	67.5 ± 3.3	15.9 ± 1.1
6	70.0 ± 0.2	16.2 ± 1.8	73.5 ± 7.2	17.4 ± 0.8

**Table 4:** Mean and standard deviation (SD) for maximal dynamic strength tests (1RM; kg) and muscular endurance test (RMs; rps) for the different exercises (LE = leg extension; LC = leg curl; HLP = horizontal leg press; BP = bench press, SR = seated row) for each training group (G% and GPE), over training program (weeks 0, 4, 8, and 12).

	Week 0 Mean ± SD	Week 4 Mean ± SD	Week 8 Mean ± SD	Week 12 Mean ± SD	Time factor	Group Factor	Time × Group Interaction
1RM LE (kg) G% GPE	23.50 ± 3.55 ^a^ 24.50 ± 3.08 ^a^	25.25 ± 3.28 ^b^ 26.08 ± 3.03 ^b^	26.67 ± 2.93 ^c^ 27.28 ± 2.49 ^c^	28.78 ± 3.67 ^d^ 28.48 ± 3.30 ^d^	< 0.001	0.659	0.272
1RM LC (kg) G% GPE	14.67 ± 3.23 ^a^ 14.31 ± 3.09 ^a^	16.79 ± 2.63 ^b^ 16.48 ± 2.99 ^b^	18.23 ± 2.11 ^c^ 17.68 ± 2.91 ^c^	19.71 ± 1.96 ^d^ 18.56 ± 2.28 ^d^	< 0.001	0.565	0.454
1RM HLP(kg) G% GPE	64.58 ± 12.02 ^a^ 61.33 ± 10.46 ^a^	70.75 ± 11.04 ^b^ 70.92 ± 10.09 ^b^	77.74 ± 14.37 ^c^ 82.74 ± 15.66 ^c^	89.96 ± 16.37 ^d^ 95.75 ± 18.33 ^d^	< 0.001	0.715	0.083
1RM BP (kg) G% GPE	28.35 ± 5.22 ^a^ 26.63 ± 5.49 ^a^	31.45 ± 6.00 ^b^ 29.71 ± 5.25 ^b^	32.75 ± 6.33 ^c^ 33.13 ± 5.72 ^c^	36.11 ± 7.23 ^d^ 35.71 ± 5.28 ^d^	< 0.001	0.721	0.120
1RM SR (kg) G%$$GPE	40.64 ± 5.92 ^a^ 41.08 ± 5.72 ^a^	44.18 ± 6.37 ^b^ 43.85 ± 5.35 ^b^	47.33 ± 7.07 ^c^ 46.08 ± 4.98 ^c^	48.69 ± 6.45 ^d^ 49.19 ± 5.27 ^d^	< 0.001	0.946	0.193
RMs LE (reps) G% GPE	8.91 ± 1.45 ^a^ 10.46 ± 2.40 ^a^			13.82 ± 1.99 ^b^ 14.92 ± 2.84 ^b^	< 0.001	0.102	0.665
RMs BP (reps) G% GPE	9.89 ± 1.45 ^a^ 10.42 ± 3.26 ^a^			17.44 ± 3.21 ^b^ 20.67 ± 4.52 ^b^	< 0.001	0.190	0.034

Note: Significance is indicated for time and group factors along with interactions between time and group (*p* < 0.05). Differences between training weeks are shown by different letters.

## Data Availability

Original dataset can be asked directly to the authors.
